# MicroRNAs Regulate Thymic Epithelium in Age-Related Thymic Involution via Down- or Upregulation of Transcription Factors

**DOI:** 10.1155/2017/2528957

**Published:** 2017-09-10

**Authors:** Minwen Xu, Xiaoli Zhang, Ruiyun Hong, Dong-Ming Su, Liefeng Wang

**Affiliations:** ^1^First Affiliated Hospital, Gannan Medical University, Ganzhou 341000, China; ^2^Department of Biotechnology, Gannan Medical University, Ganzhou 341000, China; ^3^Institute for Molecular Medicine, University of North Texas Health Science Center, Fort Worth, TX 76107, USA

## Abstract

Age-related thymic involution is primarily induced by defects in nonhematopoietic thymic epithelial cells (TECs). It is characterized by dysfunction of multiple transcription factors (TFs), such as *p63* and *FoxN1*, and also involves other TEC-associated regulators, such as *Aire*. These TFs and regulators are controlled by complicated regulatory networks, in which microRNAs (miRNAs) act as a key player. miRNAs can either directly target the 3′-UTRs (untranslated regions) of the TFs to suppress TF expression or target TF inhibitors to reduce or increase TF inhibitor expression and thereby indirectly enhance or inhibit TF expression. Here, we review the current understanding and recent studies about how miRNAs are involved in age-related thymic involution via regulation of TEC-autonomous TFs. We also discuss potential strategies for targeting miRNAs to rejuvenate age-related declined thymic function.

## 1. Introduction

The ubiquitous and abundant existence of small noncoding microRNAs (miRNAs) in worms, plants, and animals play an important role in the regulation of gene expression, which primarily occurs at posttranscriptional levels via cleavage and/or translational repression of messenger RNAs (mRNAs) [1]. Ample evidence shows that miRNAs control a wide range of developmental and physiological pathways, including cell proliferation [2], differentiation [3], and apoptosis [4]. Thus, deregulation of miRNAs will cause certain developmental obstructions, deficiencies, and even the onset of diseases [5]. The miRNA regulation is also engaged in several aspects of thymic biology [6], which are critical for T lymphopoiesis. The entire process of thymus organogenesis, maturation, and age-related involution is tightly regulated by transcription factors (TFs) [7], which, in turn, could be regulated at posttranscriptional level by miRNA genes [8, 9]. The thymus is composed of mainly hematopoietic thymocytes and nonhematopoietic thymic epithelial cells (TECs). TECs play a key role in supporting thymocyte development and controlling thymic aging. Although thymocytes possess their own transcription factors (TFs) to control their autonomous activities, many thymic activities during thymic development and aging can be regulated by known TFs in TECs, such as the *p63* and *FoxN1* [10–13]. However, regulation of these TFs remains mysterious and there is limited evidence as to the mechanisms involved. Given that many miRNAs are expressed in the thymus with different expression profiles at different developmental stages, we have adequate reasons to infer that miRNAs can be responsible for the regulation of TFs which are involved in maintaining normal thymic microenvironment that supports T lymphocyte development and controls age-related thymic involution. In this review, we focus on recent research progress which helps to elucidate how miRNA genes regulate TEC homeostasis and aging by affecting TEC-specific TFs. This summary about miRNA-mediated regulation will provide us some new insights into the regulatory networks underlying the construction and maintenance of the thymic microenvironment during thymic aging and even provide potential strategies for rejuvenating the function of the aged thymus.

## 2. Thymic Stromal Cell Homeostasis, Thymic Aging, and Transcriptional Regulation

The thymus is one of the most important organs in animal life. It generates T lymphocytes and supports the cellular immune system involved in the activities of antitumor, antivirus, and anti-intracellular infection, as well as in the establishment of self-tolerance to prevent autoimmune diseases. The thymus is also one of the most active organs, as it undergoes organogenesis (cell migration, proliferation, and differentiation), development (proliferation, differentiation, and cell apoptosis), and age-related involution (cell senescence and apoptosis) [14]. The aging process in the thymus starts in early adolescent years, and the typical thymic aging phenotype is thymic involution [15, 16].

There are two progenitor cell types in the thymus, hematopoietic thymocytes and nonhematopoietic TECs [17]. They interact and regulate each other in thymic development, homeostasis, and aging. Both cell types undergo a stepwise or sequential developmental process [18, 19]. In principle, TECs play a primary role in constructing the three-dimensional thymic meshwork and maintain the thymic microenvironment to support T cell development. TEC development and homeostasis are critical for determining thymic organogenesis prenatally and also regulate thymic involution during aging [20, 21].

Age-related thymic involution does not only reduce the output of naïve T cells but also increase the release of self-reactive T cells from the thymus [22]. These age-related changes create the basis for many age-related diseases, such as immunosenescence, chronic inflammatory diseases, including cardiovascular and neurodegenerative diseases, autoimmunity, and cancer. Age-related thymic involution appears to be a defect primarily associated with TECs [23]. TEC development and homeostasis are very meticulous processes controlled by complex regulatory networks during thymus organogenesis, homeostasis, and aging [24], which involved multiple signaling pathways and cellular interactions. Transcription factors *FoxN1* and *p63* are crucial for TEC development. In the thymus, *FoxN1*, which plays an important role in TEC survival and differentiation [25, 26], promotes differentiation of thymic epithelial progenitor cells into functional medullary thymic epithelial cells (mTECs) and cortical thymic epithelial cells (cTECs) during organogenesis [27, 28] and maintains postnatal TEC homeostasis [29, 30]. The transcription factor *p63* plays a crucial role for the epithelial development in several tissues, such as thymus and epidermis [31], and is essential for the proliferative potential of thymic epithelial progenitor cells [31, 32]. There are two *p63* isoforms: one containing an N-terminal transactivation domain, named TAp63, while the other lacking this domain is named ΔNp63. ΔNp63 and FoxN1 are both highly expressed in the fetal thymus [11, 33], but, in the adult thymus, both FoxN1^+^ and ΔNp63^+^ TECs are decreased with age [10, 34, 35]. So far, the mechanism underlying this decline is largely unknown.

Another very important transcription factor expressed in mTECs is the autoimmune regulator (*Aire*) gene; the expression of which is also declined with age [36, 37]. Although it is uncertain whether *Aire* functions to regulate the differentiation of immature TECs [38], its role in regulating clonal deletion of self-reactive T cells is definite [39, 40]. Although thousands of target genes induced by *Aire* have already been identified and well characterized, the regulation of *Aire* gene itself remains elusive. Recently, many regulators which might act upstream of *Aire* have been identified [41]. For example, a *FoxN1*-Cre-induced ablation of DGCR8, a component of the miRNA-specific microprocessor complex, eliminated Aire expression in TECs, implying a potential role of miRNA in the regulation of *Aire* gene, since DGCR8 participates in the pri-miRNA to pre-miRNA processing [42, 43]. However, the specific miRNAs involved in *Aire* regulation and the mechanisms by which they modulate Aire expression need further investigation.

## 3. A Fine-Tuning Role of miRNAs in Thymic Epithelial Cell Homeostasis

**Figure 1:**
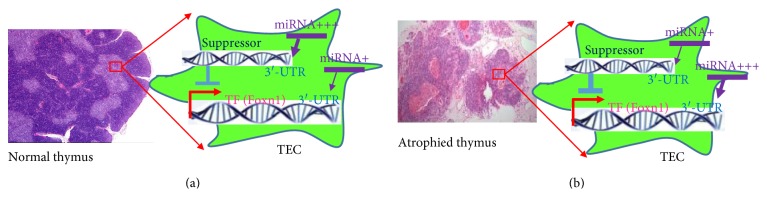
miRNA fine-tune age-related thymic involution through regulation of TEC-autonomous transcription factors. (a) Under normal conditions, a given TF (such as Foxn1) is fine-tuned by miRNAs at its 3′-UTR sites; meanwhile, the TF is also potentially regulated by its suppressive factors, which is also fine-tuned at their 3′UTR sites by miRNAs. The regulatory networks coregulate TF expression; (b) in the aged condition, some miRNAs, which directly suppress TFs, are potentially increased (from + to +++). At the same time, other miRNAs, which suppress TF suppressors, may be decreased (from +++ to +), which results in enhancement of the TF-suppressor expression which inhibit TF expression. The consequence of this combination is that the TF level is decreased. If the TF for TEC homeostasis is decreased during aging, age-related thymic involution takes place.

The miRNAs are posttranscriptional regulators involved in transcriptional repression or enhancement. Notably, a single miRNA can regulate multiple genes and a single gene can be regulated by multiple miRNAs [44]. Gene expression can be turned on either by TFs or indirectly by downregulation of other suppressive genes [45]. Expression of TFs can be suppressed either by miRNAs at their 3′-UTRs or by other suppressive genes. The suppressive genes can also be regulated by miRNAs [46]. A diagram of this regulatory network is schematically shown in Figure 1. Therefore, miRNAs play a fine-tuning role by targeting mRNAs of both TFs (direct suppression) and TF suppressors (indirect enhancement) for cleavage, translational repression, or chromatin modification [47–49]. miRNAs function in a wide range of biological process including developmental regulation [50–52], hematopoietic cell lineage determination [53–55], cellular proliferation and death/apoptosis [56–61], fat metabolism [62, 63], neuronal patterning in nematodes [64, 65], chemosensory neurons asymmetric expression [64, 66], and oncogenesis [67–70].

Since expression of miRNAs is tightly related to tissue differentiation stages [71] and miRNAs can function to prevent cell division and drive terminal differentiation [72], miRNAs are very likely to be involved in TEC differentiation-driven thymic development and thymic involution [73]. For a given gene, its expression could be directly suppressed by some miRNAs or activated indirectly via miRNA-mediated inhibition of its upstream suppressor (Figure 1). Therefore, a mixed miRNA pool, instead of a single miRNA, is more likely to orchestrate the regulatory network involved in thymic development and aging. Within a given miRNA pool, some miRNAs may suppress certain genes, while others may suppress inhibitory genes to indirectly turn on the suppressed/silent genes. Therefore, the complicated and intricate regulatory network in the thymus can potentially be regulated for development and rejuvenation by a mixed miRNA pool, rather than by a single miRNA.

As expected, recent studies have demonstrated the role of miRNAs in TEC biology. Cortical TECs (cTECs), immature medullary TEC^low^ (mTEC^low^), and mature mTEC^high^ cells were used for miRNA microarray analysis, which demonstrated that the miRNA expression profile changes as the cell matures [74]. When the entire miRNA pool was abolished in TECs by conditionally deleting Dicer, which is the miRNA maturation enzyme responsible for cleaving the pre-miRNA to the miRNA duplex, the apoptosis of mTECs was induced and cTECs failed to impose efficient positive selection. Thymic cellularity was decreased in the Dicer conditional knockout mice, resulting in the inability to maintain a regular thymic microenvironment. Additionally, T cell phenotypes were altered, including reduced naive CD4^+^ and CD8^+^ T cells, and increased CD8^+^ effector (CD44^hi^CD62L^low/−^) and central memory (CD44^hi^CD62L^hi^) T cells, and T lymphopoietic activity was diminished [42, 75].

To further understand the function of canonical miRNAs in TECs, DGCR8 was specifically deleted in TECs using a *Cre-LoxP* system (termed *Dgcr8*^ΔTEC^) [43]. It was found that DGCR8 is critical for maintaining the proper expression of *Aire* and its ablation is associated with a disruption in the overall architecture of the thymic medulla. Furthermore, deficiency of the entire pool of miRNAs due to DGCR8 deletion in TECs caused a breakdown in central tolerance [43], which is normally established in the medulla through mTEC-mediated negative selection and thymic regulatory T cells (Treg) generation. The *Dgcr8*^ΔTEC^ mice showed a significant loss of Aire^+^ mTECs, combined with an expansion of self-reactive CD4^+^ T cells. In addition, autoantibodies and autoimmune uveitis were generated in immunized *Dgcr8*^ΔTEC^ mice when compared with littermate controls [43].

## 4. miRNAs Play a Role in Thymic Epithelial Cell Development and Homeostasis by Regulating Critical Transcriptional Factors

As mentioned above, *FoxN1* acts as a key regulator of TEC development and differentiation in the fetal and adult thymus, and miRNAs can regulate TEC development and differentiation by directly or indirectly targeting *FoxN1* gene (Figure 1). There are four reports providing evidence to confirm this point of view.

Firstly, using a *miR-205^fl/fl^:FoxN1-Cre* mice to delete miR-205 in all TECs in the thymus, Hoover group demonstrated that miR-205 plays an important role in supporting T cell development following high-dose inflammatory perturbations, because conditional ablation of miR-205 caused a severe thymic hypoplasia and delayed T cell recovery, accompanied with gene expression changes in chemokine/chemokine receptor pathways, antigen processing components, and WNT signaling system [76]. Hoover group also found that miR-205 is highly expressed in both cTECs and mTECs but is largely dispensable for thymus recovery in response to low-level inflammation [73, 77]. Compared to the *miR-205^fl/fl^:FoxN1-Cre* conditional knockout mice, *FoxN1* expression levels were 2-fold higher in *FoxN1-Cre* mice. This expression change was also confirmed using fetal thymic organ culture prepared from E14.5 (gestation at 14.5 days) embryos from wild type and *miR-205^fl/fl^:FoxN1-Cre* mice. The results suggest that miR-205 is required for FoxN1 expression and epithelial cell function in fetal organogenesis and adult homeostasis following inflammatory perturbations [76]. Furthermore, incubation with miR-205 mimics (called agomirs) restored FoxN1 levels in the fetal thymic organ culture model. MiR-205 agomirs also increased the levels of *ccl25* and *stem cell factor (SCF)*, which are downstream targets for FoxN1. MiR-205 regulates *FoxN1* levels in TECs probably by promoting the degradation of mRNAs whose products suppress *FoxN1* expression (diagramed in Figure 1, indirect impact). The authors tried to assess whether 3′-UTRs in any of nineteen candidate genes had 3 or more predicted miR-205 binding sites, in order to find genes that impact *FoxN1* [76]. In addition to miR-205, miR-18b and miR-518b were also found to affect FoxN1 by suppressing its expression, potentially through directly targeting *FoxN1* 3′-UTRs (diagramed in Figure 1, direct impact).

In the second approach, Kushwaha et al. performed miRNA profiling of bone morphogenetic protein-2-treated NT2/D1 cells using the Agilent Human V2 miRNA v.10.1 array and screened out two miRNAs, miR-18b and miR-518b, which directly bind to *FoxN1* 3′-UTRs and inhibit *FoxN1* expression [78]. Interfering with these two miRNAs separately or simultaneously can increase *FoxN1* gene expression. When these two miRNAs were overexpressed separately or simultaneously, FoxN1 expression was downregulated. These results demonstrate that miR-18b and miR-518b are upstream controllers of *FoxN1* in TECs [78]. Thirdly, miR-22 is also a posttranscriptional regulator which directly represses *FoxN1* [9]. In a TRE-miR-22 mouse model (*K14-rtTA/TRE-miR-22* double transgenic mice), miR-22 overexpression in the skin promoted the anagen-to-catagen transition, inhibited keratinocyte expansion and differentiation, and enhanced hair follicle apoptosis. Since hair development is regulated by multiple hair differentiation regulators, including *Dlx3*, *Hoxc13*, *FoxN1*, and *Lef1*, miR-22 potentially directly targets these genes [9]. Given that miR-22 impacts epithelial cell development in the skin and might regulate *FoxN1*, a logical assumption is that miR-22 is likely to control the function of thymic epithelial cells. Finally, there was a recent report in which miR125a-5p, whose expression is increased in the aged thymus, was found to negatively regulate *FoxN1* expression in the aged thymus [79].

Transcription factor Trp63, a homolog of the tumor suppressor p53, is critical for the development of epithelial tissues, including the thymus [80]. The *p63-FoxN1* regulatory axis has been shown to regulate postnatal TEC homeostasis in Su group's work [10], but the study failed to identify the upstream effector responsible for regulating this axis. It has been reported that a number of miRNAs play an important role in epidermal cell proliferation and homeostasis by targeting *p63* [81–84], implying that these miRNAs may play a role in thymic development.

The *p63* gene functions as an essential regulator of stem cell maintenance in stratified epithelial tissues and is also a target of some miRNAs. For example, miR-203 has an immediate and long-term impact on epidermal cell proliferation by directly regulating *p63* [85–88]. MiR-203 was reported to promote epidermal differentiation by restricting proliferative potential and inducing cell cycle exit through directly repressing *p63* [88]. To support that, Jackson group used established keratinocytes from *K14-rtTA/pTRE2-miR-203* double positive skin and found that miR-203 is closely correlated with the epidermal differentiation in a spatiotemporally specific manner by both immediate inhibition of cell cycle progression and long-term inhibition of stem cell self-renewal [85]. They also identified a pool of miR-203-targeted genes using a genome-wide approach. These miR-203-targeted genes, including *p63*, *Msi2*, and *Skp2*, play a coregulatory role that is crucial for driving cell cycle exit and restricting proliferative potential [85]. Furthermore, Chikh et al. demonstrated that the inhibitory apoptosis-stimulating protein of p53 (iASPP), a member of the apoptosis-stimulating protein of p53 (ASPP) family, represses *p63* expression through miR-574-3p and miR-720. They found that iASPP is required for the homeostasis of epithelia [89]. MiR-720 and miR-574-3p were found to be upregulated as a consequence of iASPP silencing using an Agilent microRNA profiling assay. When coexpressed with a luciferase reporter gene containing the 3^'^-UTR of human p63, both MiR-720 and miR-574-3p significantly reduced luciferase activity. Use of antagomirs for miR-574-3p and miR-720 in keratinocytes restored ΔNp63 endogenous protein levels in sh-iASPP cells. Furthermore, using antagomirs for miR-574-3p and miR-720 can both prevent the ΔNp63 downregulation typically observed during primary keratinocyte differentiation [89]. In addition, miR-130b has been reported to directly repress ΔNp63 expression in keratinocyte senescence [84].

On the other hand, p63 can regulate the expression of some miRNAs. TAp63 binds to and transactivates the Dicer promoter and suppresses metastasis through the regulation of Dicer and a number of specific miRNAs, including miR-130b [90]. ΔNp63 in epidermal cells is a transcriptional regulator of DGCR8, which localizes to the cell nucleus and is required for miRNA processing [91]. Further, p63 mediated cell cycle progression in epidermal cells by directly repressing miR-34a and miR-34c [92]. Many miRNAs, such as miR-192/215, miR-107, miR-96,132, and miR-145, are known transcriptional targets of p63 [46, 93]. Wu group has elucidated multiple p63-regulated miRNAs' (miR-17, miR-20b, miR-30a, miR-106a, miR-143, and miR-455-3p) roles in the onset of keratinocyte differentiation [81]. It should be noted that all these experiments were conducted in skin epithelial cells, and therefore no direct evidence has been found yet to show that miRNA regulation on p63 is also engaged in thymic development and aging. Although skin epithelial cells share many similarities with TECs and these findings can provide a shortcut to study miRNA regulation in TECs, subsequent experiments in TECs are still required.

*Aire* gene is a transcription factor that controls expression of peripheral tissue antigen (PTA) genes in mTECs. Aire controls hundreds or even thousands of PTAs and has been proposed to function as a nonclassical TF based on the fact that the gene does not have many DNA-binding sites for direct interaction [94]. As for the regulation of *Aire*, specific miRNAs, such as miR-29a, in TECs play a key role. Deletion of miR-29a resulted in a progressively decreased expression of *Aire* and *Aire*-dependent genes in a miR-29a null mutant mouse model [74]. Additionally, miR-220b may act as a regulator for *Aire* gene translation, since mutation in miR-202R significantly reduced the level of Aire protein [95]. Although there is insufficient evidence that *Aire* expression is regulated by miRNAs, *Aire* has been shown to control 30 Aire-dependent miRNAs. Eighteen of these 30 miRNAs were upregulated, and the rest were downregulated in *Aire*-silenced thymic mTECs [96], strongly suggesting that these miRNAs are under the control of *Aire.* Therefore, Aire might function as an upstream controller of these miRNAs, which in turn, plays a potential role in the control of PTAs in mTECs [42, 74, 96, 97]. Microarray profiling of TEC subpopulations showed that series of miRNAs were significantly upregulated during terminal mTEC differentiation. For example, miR-124, miR-129, miR-202, miR-203, miR-302b, and miR-467a were expressed at two- to tenfold higher levels in the mTEC^high^ than in the mTEC^low^ (expression levels were all normalized to MHC-II surface expression levels) both in mouse and human thymus. The mTEC^high^ population can be further divided into Aire^−^ and Aire^+^ subsets, and the above-mentioned miRNAs were all downregulated in Aire^+^mTEC^high^ compared to Aire^−^mTEC^high^, with the exception of miR-302b, suggesting a mutual regulatory relationship between Aire and miRNAs during mTEC maturation. It was further demonstrated that miR-202 was upregulated in both immature and mature mTECs of *Aire* null mutants, while miR-129, miR-499, and miR-302b were significantly downregulated in mature mTECs of *Aire* null mutants compared to wild type mice [74]. To determine which miRNA controls PTAs in the mTECs and whether Aire expression levels could affect these interactions, Oliveira group constructed miRNA-mRNA interaction networks and found that miRNA let-7b interacted with the PTA mRNAs and confirmed the existence of a link between Aire and miRNAs in controlling the promiscuous gene expression pattern in mTECs [94].

## 5. Potential Strategies to Rejuvenate Age-Related Declined Thymic Function by Targeting miRNAs with Agomirs and Inhibitors

Although the mechanism of thymic involution has not been fully understood yet, the role played by miRNAs in this process cannot be ignored [98, 99]. For example, Guo group demonstrated that miR-181a-5p expression was increased in aged TECs, which might contribute to age-related thymic involution through downregulating the phosphorylation of Smad3 and blocking the activation of the TGF-*β* signaling [98]. WNT signaling in thymic epithelia is essential for normal thymus development and function [100] and was suppressed in the senescent human thymus [99]. Studies compared the difference in miRNA expression between old (70-year-old men) and young (<10-month-old newborns) thymus and found that miRNAs, such as miR-25, miR-7f, and miR-134, which are known modulators of the WNT pathway, were also altered [99]. Since TEC development and homeostasis are mostly controlled by *p63*, *FoxN1*, and *Aire*, miRNAs associated with these genes would be potential targets of therapeutic value. Targeting miRNAs with mimics or inhibitors is a potential strategy to rejuvenate age-related declined thymic function. In one of our published reports, we found that miRNA pools from young and aged thymus have different spectrums [79]. The strategy to rejuvenate age-related declined thymic function would be to suppress upregulated miRNAs and promote downregulated miRNAs in the senescent TECs. We hypothesize that a mixed pool of miRNA is involved in the regulation of age-related thymic involution. Therefore, multiple combinations of synthesized miRNA mimics (agomirs) targeting the downregulated miRNAs and miRNA inhibitors (antagomirs) against the upregulated miRNAs are probably the best solution to restore the age-related declined thymic function.

Thymic atrophy is attributed to increased age-related chronic inflammation, and suppressing this inflammation may alleviate thymic atrophy or restore thymic function [101]. Since miRNAs also control inflammation reactions, this might provide another approach to rejuvenating age-related thymic involution. For example, miR146a was reported to suppress inflammation, miR155 was reported to promote inflammation, and the absence of miR146a [34, 102], or upregulation of miR155 [103–105], promotes chronic inflammation with age. Furthermore, miR146a and miR155 counterregulate the immune response during chronic inflammation. Thus, combinational application of miR146a-agomir and miR155-antagomir might attenuate age-related atrophied thymic inflammation, thereby improving central immune tolerance generation.

## 6. Summary

In conclusion, miRNAs play a role in fine-tuning multiple transcription factor (TF) expression in TECs and thereby have a significant impact on thymus organogenesis, maturation, and involution at a posttranscriptional level. We reviewed recent progresses in studying the potential function of miRNAs in age-related thymic involution. Apparently, TEC development, homeostasis, and involution are very complicated processes each with a comprehensive regulatory network. Without a doubt, transcription factors *p63*, *FoxN1*, and *Aire* should be the primary targets for rejuvenating age-related declined thymic function. Modulation of the miRNA levels for regulating these TFs in the aged thymus via synthesized miRNA mimics (agomirs) or miRNA inhibitors (antagomirs) might provide an efficient approach for rejuvenating age-related thymic involution. Although current evidence is still insufficient for explaining how miRNAs regulate these TEC-autonomous TFs and subsequently induce thymic involution, we hope this review will help to summarize previous studies and guide future work towards discovering potential miRNA candidates for therapeutic targets.

## Abbreviations

miRNA: MicroRNA
TEC: Thymic epithelial cell
cTEC: Cortical thymic epithelial cell
mTEC: Medullary thymic epithelial cell
TF: Transcription factors
Aire: Autoimmune regulator
UTR: Untranslated region
ASPP: Apoptosis-stimulating protein of p53
iASPP: Inhibitory of ASPP
PTA: Peripheral tissue antigen.
